# Mapping the Knowledge Structure of Research on Patient Adherence: Knowledge Domain Visualization Based Co-Word Analysis and Social Network Analysis

**DOI:** 10.1371/journal.pone.0034497

**Published:** 2012-04-05

**Authors:** Juan Zhang, Jun Xie, Wanli Hou, Xiaochen Tu, Jing Xu, Fujian Song, Zhihong Wang, Zuxun Lu

**Affiliations:** 1 School of Public Health, Tongji Medical College, Huazhong University of Science and Technology, Wuhan, Hubei Province, China; 2 Liyuan Hospital, Tongji Medical College, Huazhong University of Science and Technology, Wuhan, Hubei Province, China; 3 School of Humanities, Henan University of Traditional Chinese Medicine, Zhengzhou, Henan Province, China; 4 Norwich Medical School, Faculty of Medicine and Health Science, University of East Anglia, Norwich, United Kingdom; Public Health Agency of Barcelona, Spain

## Abstract

**Background:**

Patient adherence is an important issue for health service providers and health researchers. However, the knowledge structure of diverse research on treatment adherence is unclear. This study used co-word analysis and social network analysis techniques to analyze research literature on adherence, and to show their knowledge structure and evolution over time.

**Methods:**

Published scientific papers about treatment adherence were retrieved from Web of Science (2000 to May 2011). A total of 2308 relevant articles were included: 788 articles published in 2000–2005 and 1520 articles published in 2006–2011. The keywords of each article were extracted by using the software Biblexcel, and the synonym and isogenous words were merged manually. The frequency of keywords and their co-occurrence frequency were counted. High frequency keywords were selected to yield the co-words matrix. Finally the decomposition maps were used to comb the complex knowledge structures.

**Results:**

Research themes were more general in the first period (2000 to 2005), and more extensive with many more new terms in the second period (2006 to 2011). Research on adherence has covered more and more diseases, populations and methods, but other diseases/conditions are not as hot as HIV/AIDS and have not become specialty themes/sub-directions. Most studies originated from the United States.

**Conclusion:**

The dynamic of this field is mainly divergent, with increasing number of new sub-directions of research. Future research is required to investigate specific directions and converge as well to construct a general paradigm in this field.

## Introduction

Patient adherence to treatment is crucial to achieving expected treatment outcomes, as “Drugs don't work in patients who don't take them” [1]. Inadequate adherence increases the risk of treatment failure and relapse and wastes health care resources [2]–[4]. Patient nonadherence to prescribed regimens is common. Only 50% of the patients suffering from chronic diseases adhere to the prescribed treatment [4]. The proportion of treatment adherence is even lower in developing countries [4].

The problem of patient nonadherence has been widely recognized by health service providers and health care researchers. However, a systematic and comprehensive understanding of this field is required about several relevant questions. What research on adherence has been conducted? What are the core themes of existing research of the field? And what further research is required? All these questions are essential for us to develop effective measures to deal with inadequate adherence in research and practice. Literature overview and knowledge domain visualization (KDViz) [5], [6] are two information techniques to answer the above questions by drawing an exhaustive picture of the field.

KDViz is a computer-supported information processing technology that can reveal the visual appearances of data objects of scientific literatures (such as authors, keywords) and their relationships. The relationships between objects are expressed in two-dimensional or three-dimensional knowledge landscape, in order to realize the visualization on intellectual structure of the knowledge domain [5], [6]. It can effectively amplify human cognition to comprehend large amounts of data and to outline the structure and evolution of a scientific field.

To visualize the structure of scientific fields, two kinds of prominent bibliometric techniques can be employed, including co-citation analysis and co-word analysis [7]–[10]. Although co-word analysis has a relatively short history [11], [12], it provides an intuitional picture of the actual content of published papers.

Co-word analysis has been used in some theoretical and empirical studies of technology foresight [8], environmental acidification [9], [13], scientometrics [14], information retrieval [15], biological safety [16], autism [17], stem cells field [18], modern engineering [19], chemical engineering [7], arts and economics [10], to explore the research topics and their relationships and changes of selected scientific fields. These studies show its practical value and advantages over literature overview, but it is rarely used in medical research.

In this paper, we use KDViz based on co-word analysis to reveal the major themes of research on treatment adherence, to probe features of the internal research structure, and to give an overview of the development in the field of treatment adherence during 2000–2011.

## Methods

### Technics

Co-word analysis is based on the assumption that a scientific field could abstract a set of signal-words to mark literature and reflect its core contents. The frequency of words occurrence in the entire body of a selected field can reflect the important themes, and co-occurrence of multiple terms in the same literature reflects the relevance of the themes to which they refer. The more frequent the co-occurrence of a pair of words in literature, the more similar the themes they indicate [5], [10], [15], [20], [21]. Keywords of scientific publications can be treated as signal-words.

Social network analysis (SNA) is the mapping and measuring of relationships among components in a system [22]. A network in SNA consists of a set of nodes and links. The nodes represent the components and the links stand for relationships between the nodes. In this paper, we structure the keywords network of research on treatment adherence, in which the nodes are the keywords while the links represent the co-occurrence of these keywords.

To understand the structure of the keyword network in literature on treatment adherence, we evaluate the location of keywords in the network by measuring the centrality of each node and the network centralization [22]. The communication between two nodes in a network can be facilitated, blocked, distorted or falsified by a node falling between them, and therefore the node between the other two nodes has a potential to control their communication. When a particular node in a group is strategically located on the shortest communication path connecting pairs of others, that node is in a central position. The centrality is defined in terms of the degree to which a node falls on the shortest path between others, and named as betweenness centrality [23].

Measures of network centralization are based on the dominance of one node. A network is central to a single node that controls its communication. The network centralization is defined as the average difference between the relative centrality of the most central node and that of other nodes. Its value ranges from 0 to 1. It is 0 for networks where the centralities of all nodes are equal, and 1 only for the wheel or star network [23].

### Study Identification

**Figure 1:**
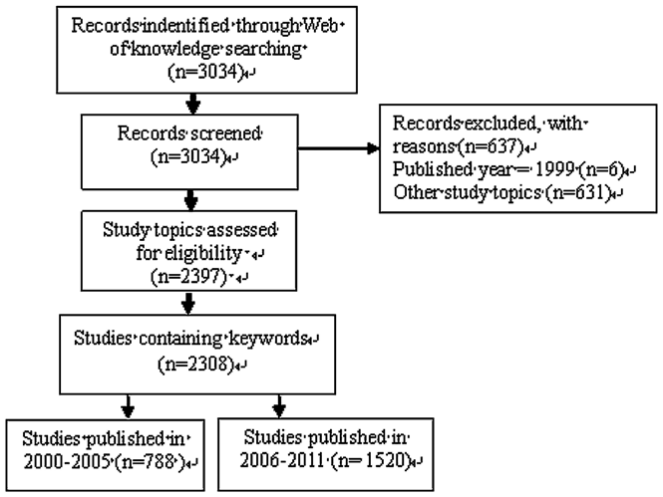
Study identification flow diagram for adherence research during 2000–2011.

We searched Web of Science for studies with the inclusion dates of January 2000 to May 2011.The primary search was based on combinations of patient and terms related to adherence (eg, adherence, compliance, nonadherence, persistence) and limited to articles. Studies focusing on patient adherence to medical regimens were included. A study was excluded if (1) the article was published before 2000; (2) it had no keywords; and (3) it reported healthcare workers' adherence to guidelines/criteria, or evaluated persistence of specific substances and phenomena (eg, modified T cells, bacteria, therapeutic effect). Two investigators independently screened these studies based on titles, abstracts, and, in a few cases, the full text, as described in Figure 1.

### Keywords Extraction

The extraction and analysis of keywords were carried out separately for the two time periods, 2000–2005 and 2006–2011. Multiple words with the same meaning were merged into one relevant word. For example, “adherence”, “non-adherence”, “compliance” and “non-compliance” were merged into “adherence”. The frequency of keywords and their co-occurrence frequency were counted. The frequency of a keyword is the number of occurrence of a keyword in all the included articles, and the co-occurrence frequency is the frequency of a pair of keywords occurring simultaneously. Finally, the keywords occurring more than or equal to 20 times, which were called high frequency words [24] in this paper, were selected to form the keyword co-occurrence matrix (co-words matrix). All above steps except the words standardization were done by Biblexcel (developed by Olle Persson, Inforsk, Umeå univ, Sweden; http://www8.umu.se/inforsk/Bibexcel/).

There are two types of keywords provided by the original authors or by ISI (keyword plus marked by ISI). Due to the fact that many articles have no author-recommended keywords, the keyword plus were used in this study.

### Data Analysis and Mapping

The co-words matrixes were input to the Ucinet6.212 software for social network analysis, and the keywords networks were displayed in two dimensional maps by the network visualization software NetDraw2.084. To simplify the network structure, a set of decomposition maps are constructed by different inclusive criterion of co-occurrence frequency.

## Results

Of 3034 papers obtained from the initial search, 2308 papers were taken into further analysis. A summary of the basic statistics of the two networks is given in Table 1. The numbers of total papers, total keywords and keywords with high frequency in the later period are all significantly larger than the previous period.

**Table 1:** Descriptive Statistics for Each Measure about Adherence Research in 2000–2011.

	Total papers	Paper with keywords	Total keywords	Total frequency of keywords	Keywords with high frequency	Total frequency of keywords with high frequency	Centrality of keywords			Network Centralization
							Mean±SD	Min	Max	
2000–2005	831	788	1591	5268	45(2.83%)	2067(39.42%)	1.499±3.403	0.000	22.598	21.58%
2006–2011	1566	1520	2740	11709	95(3.47)	6060(51.76)	0.739±1.793	0.000	16.257	15.68%
Total	2397	2308	3519	16986	131(3.72%)	9532(56.12%)	-	-	-	-

### Knowledge Structure of 2000–2005

**Figure 2:**
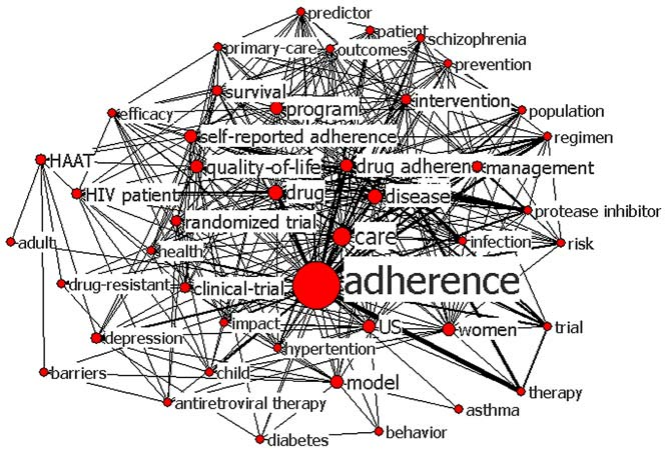
Map for keywords in adherence research, 2000–2005. The size of nodes indicates the keywords centrality, and the thickness of the lines indicates the co-occurrence frequency of keywords pairs.

**Figure 3:**
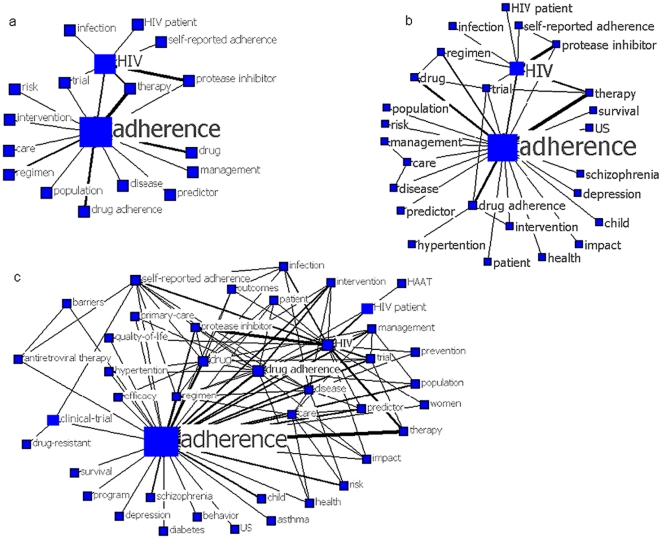
The decomposition maps for keywords in adherence research, 2000–2005. The size of nodes indicates the keywords centrality, and the thickness of the lines indicates the co-occurrence frequency of keywords pairs. The thresholds of co-occurrence frequency in map a ,b and c is ≥15, ≥10 and ≥5, respectively.

Figure 2 is the keywords network showing the knowledge structure of patient adherence in studies published between 2000 and 2005. Although the dominant word in the map is always “adherence”, other words linked to “adherence” are increasingly added in a series of subnetworks (Figure 3a–c).

Figure 3a is a network included pairs of keywords that co-occured 15 times or more. This map describes the major knowledge structure of adherence literature during 2000–2005, which covers the risk, predictor, intervention and management of adherence and involves regimen, drug, disease and population. What's more, a sub-network consisting of “adherence”, “therapy”, and “HIV” indicates that the treatment adherence of AIDS patients is a major theme in this period. When the threshold is reduced to ≥10 times, the relevant new keywords are included in the ntework, e.g. “depression”, “schizophrenia”, “child”, “US” and “survival”, with more linkages established between original words, such as “drug adherence” and “intervention”, “care” and “disease”( Figure 3b). When the threshold is lowered to ≥5 times, the linkages among the existing keywords become more density, and most new words link to two old ones, such as “prevention” links to “adherence” and “HIV” (Figure 3c).

### Knowledge Structure of 2006–2011

**Figure 4:**
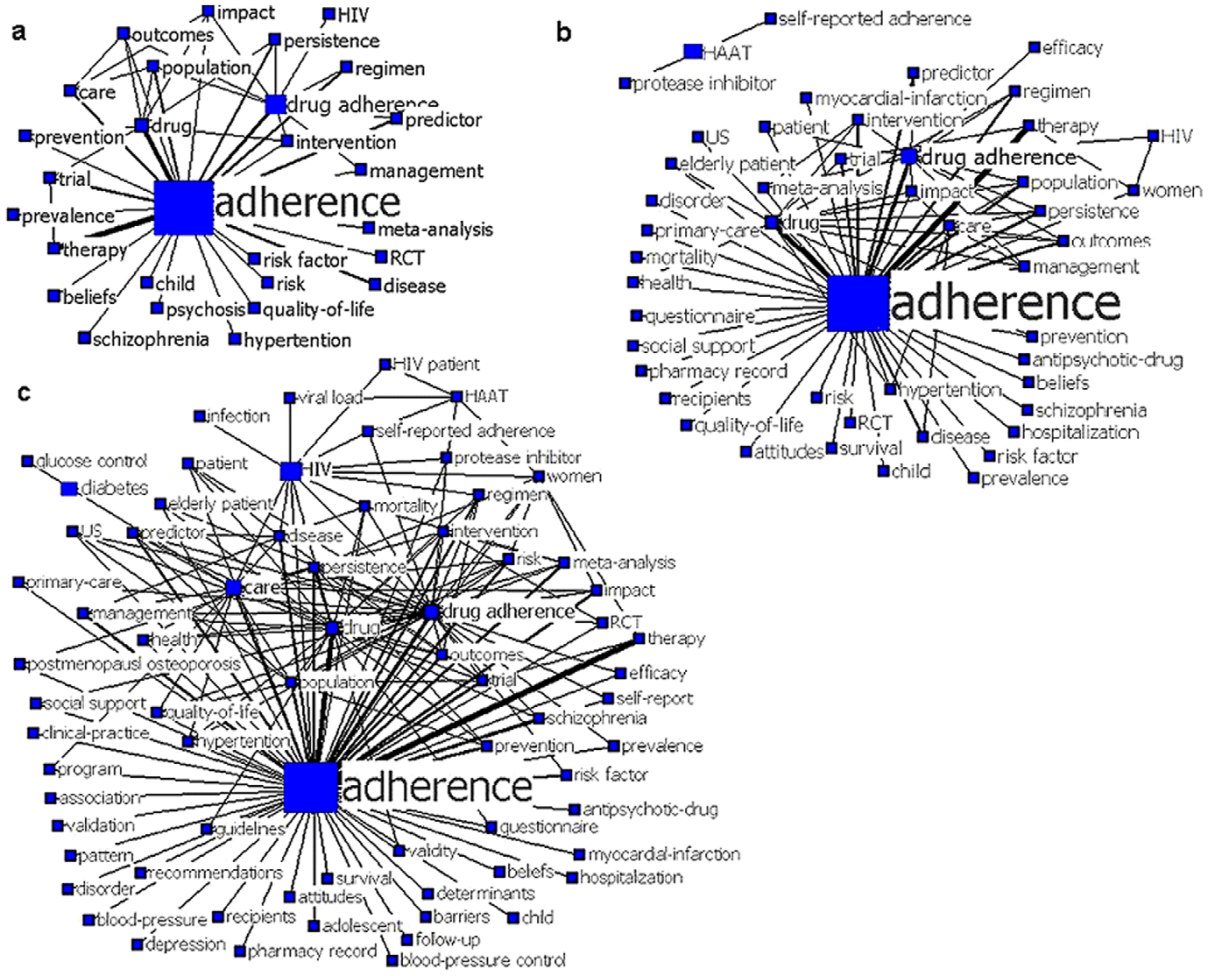
The decomposition maps for keywords in adherence research, 2006–2010. The size of nodes indicates the keywords betweenness centrality, and the thickness of lines indicates the co-occurrence frequency of keywords pairs. The thresholds of co-occurrence frequency in map a ,b and c are ≥20, ≥15 and ≥10, respectively.

There are 95 high frequency keywords extracted from the literatures during 2006–2011, and their total co-occurrence frequency is up to 6060 times. The keywords network presented by the decomposition maps is much more complex, although on the whole, it is similar to that of the first period (Figure 4). The keyword “adherence” domains the network, and the number of words and the linkages both increase gradually along with the lowered inclusion threshold. The basic structure consists of two parts. One is a sub-network formed by “adherence”, “drug adherence”, “drug”, and “care”, which suggests the research focuses of this period cover prediction, intervention, management and impact of adherence. The other part shows that “adherence” is linked independently to “prevalence”, “risk factor”, “risk”, indicating that prevalence and risk factor are hot topics as well (Figure 4a). When the threshold is ≥10 times, a sub-network shows that adherence to HIV medications is an important theme (Figure 4c).

Comparing the keywords lists and maps, we find that all the words except “clinical trial” in the first period are still visible in the second period but the frequency and relations have changed. Furthermore, the keywords in the first period tended to be general words, and more specific words emerged during the second period. These new words can be divided into 6 groups (Table 2).

**Table 2:** Groups of the New Words Emerged during the Second Period.

group	new words
1. Diseases and conditions	“chronic disease”, “clinical-practice”, “cardiovascular-disease”, “apnea syndrome”, “postmenopausal osteoporosis” , “recipients”, “antipsychotic-drug”, “self-management”, “glucose control”
2. Research methods and technicals	“RCT”, “meta-analysis”, “cohort”, “double-blind”, “follow-up”, “questionnaire”, “pharmacy record”, “reliability”, “validity”, “persistence”
3. Determinants or risk factors of patient nonadherence	“beliefs”, “knowledge”, “social support”, “communication”, “symptom”, “cost”, “recommendation”, “experience”
4. Patient nonadherence impacts	“impact”, “quality”, “mortality”, “hospitalization”, “cost”
5. Target population	“adolescence”, “elderly patient”
6. Other aspects	“prevalence”, “pattern”, “decision”, “clinical-practice”

### Individual Centrality and Network Centralization

Keywords centrality and network centralization are applied to analyze the network structure. In the network of the first period, the mean value of betweenness centrality of the keywords is 1.499±3.403, and the maximum value is 22.598. In the second period, the mean value of keywords centrality is 0.739±1.793, and the maximum value is 16.257.

Seen from Figure 3, “adherence” and “HIV” have the largest betweenness centrality, and play a “hub” role in the network. Without them, the network structure would be changed greatly. For example, if “HIV” is deleted, “infection” and “HIV patient” would be cut off from the network. AS new words and links of lower co-occurrence frequency emerge in the network, the centrality of “HIV” decreases relatively and that the centrality of “drug adherence” and “drug” increases. In the keywords network of 2006–2011, “adherence” still has the greatest centrality but less than that of the first period, and the number of keywords with larger centrality have changed from one (“drug adherence”) to four (“drug adherence”, “care”, “drug” and “HIV”). The difference in individual centrality is greater in the first period as compared to that in the second period. Similarly the network centralization decreases from 21.58% in 2000–2005 to 15.68% in 2006–2011. These suggest that the network of the second period is less centralized (Figure 4).

## Discussion

The number of studies on treatment adherence has been dramatically increasing because of the increased awareness of its importance in healthcare practice. Treatment adherence has become an emerging research field which requires a systematic analysis of its knowledge structure. This study integrates co-word analysis and SNA to investigate knowledge structure created by journal articles of adherence, in order to systematically examine the fundamental knowledge structure and its evolution in the twenty-first century.

Below are the three main findings from this study:

1. The number of studies on adherence has been increasing. The articles published in the last period are almost twice as the first period.
2. The research subjects become more extensive and intensive. The research themes are more general in the first period. More specific topics have emerged in the second period, including risk factors, impact and measurement of patient nonadherence. There used to be a misconception that adherence was a problem driven by patients, who should be responsible for their treatment. In fact, adherence is also influenced by social and economic factors, the health care team/system, the characteristics of the disease and types of treatments [4]. As shown in this paper, these factors have recently been emphasized in research.
3. The existing adherence studies have covered broader disease spectrum, especially the chronic diseases. But researches on adherence of patients with other diseases are not as hot as HIV, and have not become specialty themes/sub-directions. The epidemiological shift from acute to chronic diseases has rendered that health system must evolve to meet new challenges. Research and practice in this field also need to focus on the prevention and treatment of chronic diseases.

Many studies aim at the treatment adherence of patients with specific populations, such as women [25] and adolescents [26]–[28]. Adherence is a complex phenomenon affected by the interaction of many factors. It is difficult to find universally applicable interventions to improve patient adherence. To improve patient adherence, the “best” intervention strategy may often be the patient-oriented. Understanding clearly the characters of patients and factors influencing patient adherence is important for efficient adherence interventions.

There were some advances in methodological aspects, not only by applying conventional research methods, but also by developing topic-specific research techniques. Accurate assessment of adherence behavior is essential for the evaluation of interventions to improve patient adherence to treatments. There is, however, no “gold standard” for measuring adherence behavior [4], and it is unlikely that we could have a method to accurately assess adherence in all circumstances. Further studies are required to investigate valid and reliable measurements of adherence.

The network of the later period is less centralized than the former, because new keywords entered and that plentiful direct connections of keywords produced over time. These new words and relationships indicate, to a certain extent, the emergence of new themes/sub-directions in adherence research. Studies of this field are rarely of a unified model and have great differences in theories, hypotheses, experimental techniques and conditions [2], [29]–[33]. The dynamics of scientific research may be classified into two model: convergent and divergent [34]. We could conclude that adherence research has been mainly divergent.

The word “US” is the only toponym occurring in the lists of high frequency keywords of the two periods. Maybe it's related to the coverage of databases we used, because not all the papers in this field are included in Web of Science [7]. However, more adherence studies of HIV/AIDS treatment were conducted in developed countries than in developing countries [32]. A systematic review of adherence interventions to phosphate binding medication in patients with end-stage renal disease found that 79% of the original literatures came from the USA [35]. This suggests that most articles originated from the USA, as well as in most other research fields [36]–[38].

In this paper, we used co-word analysis to give an overview of the knowledge structures of research on adherence during 2000–2005 and 2006–2011. In contrast, systematic reviews mainly focus on providing pooled estimates to answer specific research questions based on rigorous research evidence [39]–[41]. When there is a lack of methodologically rigorous studies and/or there are great heterogeneity and diversity across studies, a systematic review may be replaced by a narrative review [40]. As an alternative, co-word analysis can outline a selected field more widely by focusing on the content of the literature rather than the results. In addition, this method may reduce the reliance on subjective judgment [7], [9].

The complex knowledge structures could be simplified by decomposition maps, which are consistent with the process of human understanding from coarse to fine, and overcome the disadvantages of a single threshold. A low threshold may create a long list of words and a map too complex to interpret and visualize, while a higher threshold gives a broader view of the field under study [7].

As noted in other studies, there exist limitations in the basic data used in this paper, such as the scope of the database and “indexer effect” [7]. Web of Science does not have a complete coverage of the scientific researches in adherence, but it is well received by the scientific community and its computer assisted indexing technology considerably reduces the “indexer effect” [42]. It satisfies the objective of this study to identify the general research structure and the evolution of adherence research.

In summary, adherence research is still in early experimental stages, and has great potential for further development. Future research is required to investigate specific directions and converge as well to construct a general paradigm in this field.

The KDViz technic for medical research may be a valuable complement to systematic literature review, and have unique advantages particularly in the early development stages of scientific topics. The use of KDViz method for literature analysis may provide rich reference information for the researchers and decision-maker.
